# On Inflation of the Bowels

**Published:** 1831

**Authors:** 


On Inflation of the Bowels. By Arch. Blacklock, late Surgeon, R.N.

In a preceding article of the present No. of this Journal, one of our reviewers treated the subject of "inflation of the bowels," as proposed by Mr. King, of Irvine, perhaps with a little more levity than was proper, though the value attached to the remedy, by Mr. K. was certainly set forth with rather extravagant pretensions. In the 14th No. of our Glasgow contemporary, we find another communication on the same subject, from the pen of Mr. Blacklock, of Dumfries, which induces us again to notice *artificial* inflation of the bowels as a remedial agent, though the *natural* inflation is one of the most common and distressing symptoms attendant on many diseases. But on this point we shall make a remark or two presently. So long ago as 1820, Mr. Blacklock communicated to the Editor of this Journal the fact of his readily removing intus-susceptions, (where no adhesions had taken place) in the dead body, by means of injections of air per anum. It is alluded to in our Quarterly No. for April, 1820, article "Ileus." Since that time, he and other practitioners in his neighbourhood have frequently resorted to this process, in cases of obstinate obstruction of the bowels. One of these cases was Mr. Blacklock's own son, then about three years of age, who, during nine days, remained without any passage through the bowels, notwithstanding the employment of various purgatives and numerous enemata. Recourse was ultimately had to inflation, when the little patient cried out for the commode, and had a free evacuation. After a few minutes the inflation was repeated, and was soon followed by another fæcal stool. The boy rapidly recovered, and is now living and in good health.

The great inconvenience, or indeed misery, produced by flatulence in the stomach and bowels, is, perhaps, a less valid objection to the artificial inflation now under consideration, than might at first sight appear. In the first place, it is to be remembered that the gas disengaged in the primæ viæ, during indigestion and other disordered states of the digestive organs, is very far from being identical with common atmospheric air. This has been proved by chemical analysis—but still more certainly by common observation. The gases generated, in such conditions of the alimentary canal, are not only various, but many of them appear to act as acrid, or at least deleterious poisons, during their stay in the first passages. They are more distressing, also, in proportion as they are situated higher up in the alimentary canal. In the stomach and duodenum, they are far more inimical to our feelings and health, than in the small intestines—and in the *latter*, they are infinitely more difficult to bear than in the colon. The morbid and indescribable feelings resulting from the extrication of gas in the stomach, are very imperfectly known, and not at all appreciated, in a proper manner, by medical practitioners in general. Even when the eructated gas is perfectly inodorous and tasteless, its presence in the stomach produces, in many instances, the most terrible sensations. But it is the nature of these noxious gases to keep their station resolutely in the localities where they are generated, by producing spasmodic constriction of the tubes both above and below them. They are then pent up, and their presence not always, or even often, suspected or known. These, and many other considerations which we could adduce, may lead us to suppose that the introduction of common atmospheric air into the colon, or even into the small intestines, will be unattended with the inconvenient or injurious effects resulting from aerial or gaseous products of disordered digestion. If medical men were to make experiments on their own persons more frequently than they do, their patients might be benefited and their own sphere of knowledge very much extended. It would be well worth while to try the substitution of common air for warm water, in cases of constipated bowels; for if the former fluid answers as well as the *latter*, the facility of its employment in all situations would be an immense advantage. We intend to experiment on this point of practice, and we solicit our medical brethren to do the same, and to communicate the results of their observations to the public.

---

